# Easy-to-Use Preservation and Application of Platelet-Rich Plasma in Combination Wound Therapy With a Gelatin Sheet and Freeze-Dried Platelet-Rich Plasma: A Case Report

**Published:** 2016-08-05

**Authors:** Naoki Morimoto, Natsuko Kakudo, Tsunekata Ogura, Tomoya Hara, Makoto Matsui, Masaya Yamamoto, Yasuhiko Tabata, Kenji Kusumoto

**Affiliations:** ^a^Department of Plastic and Reconstructive Surgery, Kansai Medical University, Osaka, Japan; ^b^Polymer Chemistry Division, Chemical Resources Laboratory, Tokyo Institute of Technology, Tokyo, Japan; ^c^Department of Biomaterials, Field of Tissue Engineering, Institute for Frontier Medical Sciences, Kyoto University, Kyoto, Japan

**Keywords:** gelatin sheet, platelet-rich plasma, chronic skin ulcers, freeze-dried, controlled release

## Abstract

**Objective:** Platelet-rich plasma is blood plasma enriched with platelets and contains various growth factors. Two major issues remain to be resolved in the use of platelet-rich plasma: the short biological activity after application, and the need to prepare platelet-rich plasma at each application instance. To overcome these problems, we developed a drug delivery system using gelatin hydrogel and preserved the excess platelet-rich plasma as freeze-dried platelet-rich plasma. We then applied combination treatment with a gelatin sheet and platelet-rich plasma at the first instance and freeze-dried platelet-rich plasma at the second instance in the treatment of a nonhealing wound. **Methods:** A 68-year-old woman had suffered open fracture of her right tibia 2 years prior, and a split-thickness skin graft had been applied to repair the skin defect on the right tibia. She had multiple relapse of ulcers, and the present ulcer had not healed for 2 months. After debridement, 2 mL of activated platelet-rich plasma was applied to the ulcer, and the gelatin sheet was laid to impregnate with the platelet-rich plasma, after which the sheet was covered with a polyurethane film. Thirty-three days after the first platelet-rich plasma application, the freeze-dried platelet-rich plasma was reconstituted and 2 mL of the reconstituted platelet-rich plasma was applied with a gelatin sheet. **Results:** At 14 days after the freeze-dried platelet-rich plasma application, the wound was mostly epithelized, with the rest of the wound covered with granulation tissue. **Conclusions:** These findings suggest that combination wound therapy with a gelatin sheet and freeze-dried platelet-rich plasma is a promising method for resolving issues with conventional platelet-rich plasma treatment.

Platelet-rich plasma (PRP) is blood plasma enriched with platelets, and autologous PRP contains various growth factors, such as platelet-derived growth factor (PDGF), transforming growth factor-β (TGF-β), and vascular endothelial growth factor.^1^^,^^2^ Regarding the efficacy of PRP in the treatment of chronic wounds, reports are conflicting; PRP has been reported to improve the rate of healing in many studies, whereas others have found no major differences in the healing outcomes of leg ulcers between PRP treatment groups and control groups.^3^^,^^4^

Two major issues concerning the preparation and usage of PRP in clinical practice remain to be resolved. The first issue is the short biological activity of growth factors after application in vivo. To overcome this, we developed a drug delivery system using gelatin hydrogel with an isoelectric point (IEP) of 5.0 for the sustained release of positively charged growth factors, such as PDGF and TGF-β 1. Our system proved effective for combination wound therapy with a gelatin sheet and PRP in a murine model.^5^^,^^6^ The second issue is the need to prepare PRP from the patient's peripheral blood at the time of application, as no long-term (>1 week) preservation method for PRP has yet been established. We applied our freeze-dried procedure to preserve human platelet lysates and found that the activities of the growth factors were maintained after reconstitution with normal saline solution.^7^

In the present report, we applied combination treatment with a gelatin sheet and autologous PRP at the first instance in the treatment of nonhealing wound and preserved the extra PRP as the freeze-dried PRP. We then applied a gelatin sheet impregnated with the freeze-dried PRP at the second instance and compared the outcomes.

## MATERIALS AND METHODS

The study protocol was approved by the institutional review board of Kansai Medical University (KMU no. 0649-1). PRP was prepared from 60 mL of the patient's peripheral blood using the Magellan Autologous Platelet Separator System (Medtronic Inc, Minneapolis, Minn) and its basic disposable kit. The obtained PRP was activated using CaCl_2_ (Otsuka Pharmaceutical Factory, Inc, Tokushima, Japan) as described in our previous study.^1^^,^^2^ The extra PRP was freeze-dried using a freeze-drier (EYELA FDU-2200; Tokyo Rikakikai Inc, Tokyo, Japan) in accordance with the manufacturer's instructions and stored at 4°C until use. Gelatin hydrogel sheets 12 × 3 cm in size were prepared at the Department of Biomaterial, Field of Tissue Engineering, Institute for Frontier Medical Sciences, Kyoto University, as described in our previous study.^8^

## CASE REPORT

**Figure 1:**
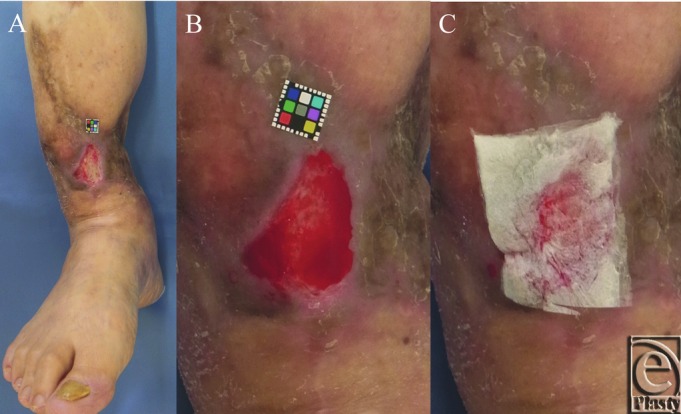
The gross appearance of the ulcer on the right leg and the application of PRP and the gelatin sheet. (a) Non-healing ulcer 26 ×16 mm in size formed in the skin graft. (b) Activated PRP was applied. (c) The gelatin sheet was applied on the wound. PRP indicates platelet-rich plasma.

A 68-year-old woman had suffered open fracture of her right tibia 2 years prior, and a split-thickness skin graft had been applied to repair the full-thickness skin defect of the open wound on the right tibia. She had multiple relapse of ulcers on the grafted skin and was admitted to our hospital. The present ulcer had not healed for 2 months at her initial visit to our hospital (Fig 1*a*). After debridement of the necrotic tissue, 2 mL of the activated PRP was applied on the ulcer (Fig 1*b*), and the gelatin sheet was laid to impregnate with the PRP (Fig 1*c*), after which the sheet was covered with the polyurethane film (OPSITE, Smith & Nephew KK, Tokyo, Japan) for 5 days.

**Figure 2:**
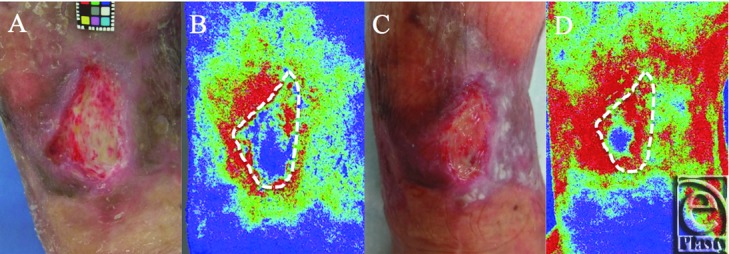
The gross appearance of the ulcer and the color-coded image of FLPI-2 before and after PRP application. The gross appearance (a) and the color-coded image (b) of the ulcer after debridement before PRP application. The mean flux was 337 in the ulcer indicated by the white doted circle. The gross appearance (c) and the color-coded image (d) of the ulcer after removing the sheet at 5 days after application. The mean flux was 625 in the ulcer indicated by the white doted circle. PRP indicates platelet-rich plasma.

The change in the blood flow of the wound bed before the application of PRP and at 5 days after removing the gelatin sheet was evaluated using a laser speckle contrast imager (Moor FLPI-2; MoorInstruments Ltd, Devon, United Kingdom). The flux value is the relative measure of the blood flow that represents the mean velocity and concentration of blood cells at a depth of about 300 μm beneath the wound surface.^9^ The granulation tissue formation was poor after debridement (Fig 2*a*), and the mean flux was 337, indicating moderate blood flow (Fig 2*b*). Five days after the application of PRP, granulation tissue began to form over the wound (Fig 2*c*) and the mean flux increased to 625 (Fig 2*d*).

**Figure 3:**
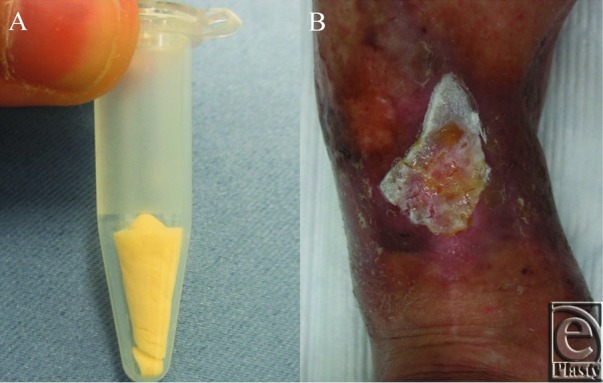
The gross appearance of freeze-dried PRP and its application to the wound. (a) Freeze-dried PRP preserved in a 1.5-mL microcentrifuge tube. (b) Freeze-dried PRP was reconstituted to the original PRP volume with the normal saline solution, and 2 mL of reconstituted PRP was applied and covered with a gelatin sheet. PRP indicates platelet-rich plasma.

**Figure 4:**
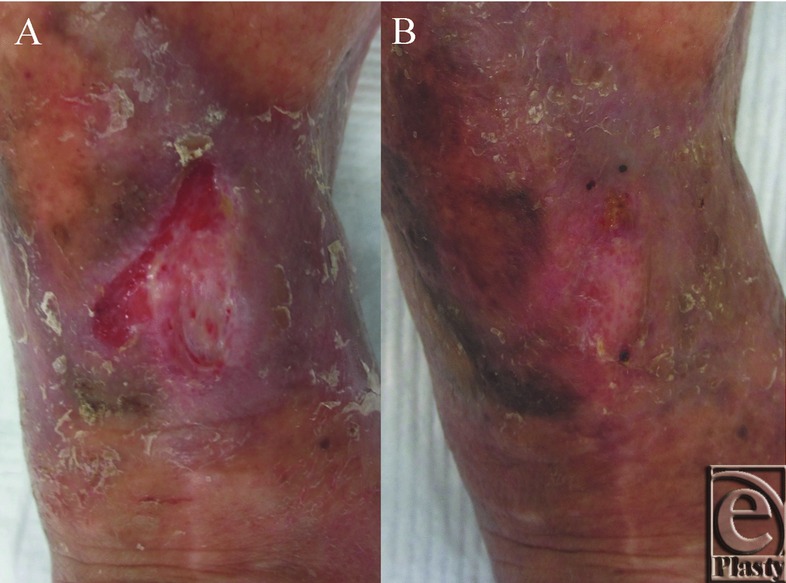
The gross appearance of the wound at 37 days (a) and 9 months (b) after the first PRP application. (a) Fourteen days after freeze-dried PRP application (37 days after the first application), more than half of the wound was epithelialized, and the rest of the wound was covered with granulation tissue. (b) The wound has no recurrent ulceration after 9 months. PRP indicates platelet-rich plasma.

Thirty-three days after the first PRP application, freeze-dried PRP (Fig 3*a*) was reconstituted, and 2 mL of the reconstituted PRP was applied, covered with the gelatin sheet (Fig 3*b*), and then covered with the polyurethane film. The film was removed 5 days after its application, and Azulene ointment (Azunol ointment; Nippon Shinyaku Co, Ltd, Kyoto, Japan) or Alprostadil Alfadex ointment (Prostandin ointment: Ono Pharmaceutical Co, Ltd, Osaka, Japan) was applied until complete epithelization was achieved. Fourteen days after the freeze-dried PRP application, the wound had mostly epithelized and the rest of the wound was covered with granulation tissue (Fig 4*a*). The wound has had no recurrent ulceration in the 9 months that have passed since the procedure (Fig 4*b*). No side effects such as inflammation or infection were observed after the application of the gelatin sheet or freeze-dried PRP during the treatment.

## DISCUSSION

Our gelatin sheet has been applied in clinical trials mainly for the controlled release of basin fibroblast growth factor (bFGF), as human recombinant bFGF (FIBRAST SPRAY; Kaken Pharmaceutical, Tokyo, Japan) has been clinically used for chronic skin ulcers since 2001, and its clinical effectiveness has been demonstrated.^10^ We also previously reported that our controlled release system of bFGF (collagen/gelatin sponge containing 10% [wt/wt] gelatin with an IEP of 5.0) showed excellent efficacy in the treatment of chronic skin ulcers. However, bFGF is approved for clinical use only in Japan and China, so we tried to apply our gelatin sheet in combination with PRP.

As for the clinical application of PRP, the preservation of PRP is an issue, since PRP must be prepared from the patient's peripheral blood at the time of application. We previously reported that PDGF-BB and TGF-β in platelet lysates could be concentrated up to 4-fold using a freeze-dried technique, and the bioactivity of PDGF-BB and TGF-β was maintained after reconstitution.^7^ In the present study, we freeze-dried the patient's extra PRP and refrigerated it until the second application with a gelatin sheet. The blood flow of the wound bed as evaluated using FLPI-2 increased after the first application, and the wound epithelization was accelerated after the second application.

To our knowledge, this is the first case report of combination therapy with a gelatin sheet and freeze-dried autologous PRP. This combination treatment providing sustained release of PRP was not only effective in maximizing the efficacy of PRP but also efficiently used excess PRP without waste. We will continue our attempts to demonstrate the efficacy and safety of this treatment in order to improve the shortcomings of a conventional PRP treatment.
